# Determinants of overweight and obesity and other cardiometabolic risks in adolescents: a Spanish longitudinal birth study

**DOI:** 10.1038/s41390-025-04273-w

**Published:** 2025-07-18

**Authors:** Manuel Lozano, Jorge Vallejo-Ortega, Natalia Marín, Llúcia González-Safont, Ana Esplugues, Maria-Jose Lopez-Espinosa, Andrea Beneito, Sandra González-Palacios, Sabrina Llop, Raquel Soler-Blasco

**Affiliations:** 1 Epidemiology and Environmental Health Joint Research Unit, FISABIO−Universitat Jaume I−Universitat de València, Valencia, Spain; 2 Preventive Medicine and Public Health, Food Sciences, Toxicology and Forensic Medicine Department, Universitat de València, Valencia, Spain; 3 Spanish Consortium for Research on Epidemiology and Public Health (CIBERESP), Madrid, Spain; 4 Foundation for the Promotion of Health and Biomedical Research in the Valencian Region, FISABIO-Public Health, Valencia, Spain; 5 Department of Nursing, Universitat de València, Valencia, Spain; 6 Multiprofessional Teaching Unit for Family and Community Care (UDMAFiC), Catalan Health Institute-Camp de Tarragona, Tarragona, Spain; 7 Grupo de Epidemiología de la Nutrición. Universidad Miguel Hernández (UMH), Alicante, Spain; 8 Alicante Institute for Health and Biomedical Research (ISABIAL), Alicante, Spain

## Abstract

**Background:**

To assess the prenatal, early postnatal and adolescent factors associated with overweight/obesity (OwO) and other cardiometabolic risk factors at age 15.

**Methods:**

Longitudinal study based on 241 participants from the INMA-Valencia cohort. Z-scores of body mass index (zBMI), waist circumference (zWC), and waist-to-height ratio (WHtR), systolic and diastolic BP (zSBP and zDBP) were evaluated at ages 4, 7, 9, 11 and 15. A cardiometabolic risk score was calculated. Covariates were collected at pregnancy, birth, and age 15.

**Results:**

At age 4, 30.7% presented overweight/obesity (zBMI > 1 SD), increasing to 46.1% at age 9, and decreasing to 29.3% at age 15 (29.3%). The proportion of high/excess central adiposity was lower than overweight/obesity at all ages. Adjusted models showed that pre-pregnancy obesity was positively associated with all cardiometabolic outcomes, except zSBP (i.e. *β* [95% CI]: 0.77 [0.25, 1.29] for zBMI, and 2.31 [0.94, 3.69] for CMR score). Smoking during pregnancy was directly related to zSBP and zBMI. Adolescent physical activity was inversely associated with WHtR, zFM, zWC, zDBP and cardiometabolic risk scores (*β* −0.65 [95% CI −0.95, −0.36]). Cereal intake and processed meat consumption were positively related to WHtR, zBMI and zWC.

**Conclusion:**

Early modifiable factors impact adolescent cardiometabolic health. This information could improve preventive interventions and policies from very early.

**Impact:**

- This longitudinal study shows how sociodemographic, clinical, and lifestyle factors influence adolescents’ cardiometabolic health from very early stages of life until adolescence.
- Maternal characteristics, such as pre-gestational obesity or tobacco consumption during pregnancy, were directly associated with cardiometabolic risk factors in adolescence, such as BMI, percentage of fat mass, and diastolic blood pressure.
- Adolescents’ intake of cereals, and processed meats, and consumption of less than five dairy meals, were positively related to an increase in cardiometabolic risk factors.
- Findings from this longitudinal study provide valuable insights for designing early-life interventions in prevention, health promotion, and adolescent health management.

## Introduction

Cardiovascular and metabolic diseases are the most common in adulthood, and the first cause of premature death and disability worldwide,^1,2^ representing a major public health issue. Excess weight and adiposity are recognised as key cardiometabolic factors, strongly associated with the development of other cardiometabolic risks, including hypertension, dyslipidemia, and insulin resistance.^3,4^ The interplay of these factors substantially increases the probability of cardiovascular and metabolic events across the lifespan.^3,5^

Nowadays, it is well established that a complex network of multiple entangled elements, such as lifestyle, environmental factors, social determinants, and genetic predisposition, influences the development of excess adiposity and other cardiometabolic risk factors.^6^ In recent decades, multiple studies support that early life factors, from foetal development through childhood and adolescence, have an impact on the development of cardiometabolic risks.^7^ This is framed in the Developmental Origins of Adult Health and Disease paradigm, which postulates that the environment during early development, including the prenatal and early postnatal stages, could increase the risk of disease throughout life.^5,8,9^ From this perspective, multiple factors and the interactions among them have been associated with the development of these cardiometabolic risk factors in childhood and adolescence periods, namely, dietary patterns,^10^ sedentarism and physical activity levels,^10–12^ screen exposure,^10,13^ sleep disorders,^10^ family and social environment,^14,15^ and social position,^15^ among others.

Additionally, some factors during prenatal and early postnatal periods, such as maternal obesity before pregnancy,^16–18^ gestational diabetes,^16^ gestational weight gain,^17^ prenatal tobacco exposure,^19^ or maternal nutrient status during pregnancy^20^ have been strongly associated with an increased risk of developing excess weight and adiposity, and other cardiometabolic risks during childhood, adolescence and adulthood.

Pre-birth cohort studies are the most suitable design to examine the relationship between prenatal and postnatal factors and the development of cardiovascular risk factors in adolescence, allowing the prospective collection of detailed and high-quality data throughout an individual’s life. The identification of critical factors risk in early development could help appropriate windows for intervention. In the present study, we aimed to describe the proportion of early postnatal and adolescent overweight and obesity, central adiposity and other cardiometabolic risk factors (systolic and diastolic BP and cardiometabolic risk score), and to study prenatal and postnatal associated factors in a Spanish pre-birth cohort.

## Materials and methods

### Study population

The participants in the study were mother-child pairs from the Childhood and Environment (INMA) Project, a multicentre birth cohort study across various geographical regions of Spain (http://www.proyectoinma.org). Subjects were participants from the Valencia area (eastern Spain). Details of the study protocol can be found in Guxens.^21^

Briefly, during 2003–2005, 855 pregnant women were recruited in their first prenatal visit at their centre of reference (La Fe Hospital, Valencia). Eligibility criteria were a minimum of 16 years of age, 10–13 weeks of gestation, non-assisted conception, singleton pregnancy, expressing the intention to undergo follow-up at the reference centre, and being able to communicate without any difficulties. After excluding those women who withdrew from the study, were lost to follow-up, and had induced or spontaneous abortions or foetal deaths, we successfully followed up with 787 women until delivery. Their children were enroled at birth and were followed up until they were 15 years old (*n* = 281, 2019–2021). The selection of participants, inclusion criteria and reasons for non-participation can be consulted in Fig. S1. The final study samples comprised: (1) mother-child pairs in whom anthropometric outcome assessment at age 15 was available (*n* = 241) and (2) mother-child pairs in whom blood samples were available to evaluate the CMR score at age 15 (*n* = 200).

The study protocol was approved by the University Hospital La Fe and the FISABIO-Public Health Ethics Committees. Informed consent was obtained from the mother (prenatal period) and either one of the parents or a legal representative (postnatal period). At the 15-year follow-up visit, adolescents signed an informed assent.

### Study variables and sources of information

#### Anthropometry assessment

Participants’ height, weight, and waist circumference (WC) were measured by trained staff during the clinical examinations in the follow-up visits (4, 7, 9, 11 and 15 years) using a standard protocol. Height was measured with a mobile stadiometer (Seca model 213). Weight and percentage of fat mass (FM) were measured using an electronic scale (Tanita model BC-351). WC was measured at the middle point between the lower margin of the last palpable rib and the top of the iliac crest after a normal expiration, using an inelastic measuring tape (Seca 201), following the World Health Organization (WHO) protocol.^22^ Two consecutive measurements of each height, weight, FM, and WC were taken, and the mean was calculated and used in further analysis. Age- and sex-specific BMI *z*-scores were calculated using the WHO Growth Reference.^23^ Overweight was defined as BMI *z*-scores +1 standard deviation (SD) and obesity +2SD. waist-to-height ratio (WHtR) was calculated using WC (cm) and height (cm) to measure central adiposity. We used the validated cut-off points proposed for the paediatric population (very low/normal trunk fat [<0.53 in boys and <0.54 in girls], high/excess trunk fat (≥0.53 in boys, and [≥0.54 in girls]).^24,25^ More information about the anthropometric assessment can be consulted in Appendix S1.

#### Other cardiometabolic risk factors evaluated

Blood samples from adolescents were collected at the 15-year follow-up visit. After separating the serum by centrifugation, samples were stored at −80 °C and transported frozen to the Biosanitary Research Institute of Granada (Spain). Concentrations of high-density lipoproteins (HDL cholesterol), low-density lipoproteins (LDL cholesterol), and glucose were analysed using a Cobas® c-311 analyser (Roche Diagnostics, Basel, Switzerland).

Blood pressure was measured during the clinical examination in the same follow-up visit using a standardised protocol (Appendix S1). Three consecutive measurements were taken by an oscillometric device (OMROM M4-I) with at least 1-min time intervals between measurements. The device is clinically validated according to the International Protocol of the European Society of Hypertension and criteria suggested by the British Hypertension Society for its use in children and adolescents.^26^ The mean of the second and third measurements of systolic and diastolic blood pressure (SBP and DBP) was calculated and used in further analysis. Median blood pressure (MBP) was calculated using the formula (SBP + DBP)/3 + SBP.

To date, no harmonised cardiometabolic risk score for adolescents has been reported in the literature, and several criteria have been used in previous research.^27^ For the present study, we used the continuous cardiometabolic risk score proposed by Fernández-Aparicio et al.^28^ We selected the cardiometabolic risk *z*-score as it presented the highest predictive power (higher area under the curve) and specificity compared with other methods (including continuous metabolic syndrome scores, principal components, and confirmatory factor analysis) studied in a population of Spanish adolescents.^28^ This score was calculated after standardising the residuals (*z*-scores) for glucose, serum triglyceride and HDL concentrations, MBP, and WC by regressing them on age and sex to account for age- and sex-related differences. For WC and MBP, height was also included in the regression to account for this variable. For the standardisation calculation, the study population was used as the reference population. The cardiometabolic risk score was calculated as the sum of the *z*-scores of these variables. zHDL concentrations were introduced into the formula multiplied by −1 (due to their inverse relationship with metabolic risk). Higher scores indicate higher cardiometabolic risk.

#### Covariates

##### Sociodemographic, lifestyle and dietetic variables during pregnancy and birth

Pregnant women filled out two questionnaires, administered by trained interviewers, at early (mean: 12.6 weeks of gestation) and late (32.1 weeks of gestation) pregnancy. The covariates considered in the present study were the following (more information in Table S1): maternal and paternal BMI before pregnancy, maternal and paternal education level, maternal and paternal employment status at pregnancy, and parental social class during pregnancy—defined by maternal or paternal occupation—maternal tobacco consumption during the 1st trimester and the whole pregnancy, parity, maternal total physical activity during pregnancy expressed as overall metabolic equivalent of task levels (continuous),^29^ weight gain during pregnancy according to the Institute of Medicine guideline Recommendations,^30^ any glucose impairment during pregnancy (including impaired glucose tolerance, gestational diabetes, or a diabetes diagnosis before pregnancy).

Information regarding the perinatal period was obtained from medical records: child’s sex, preterm (<37 weeks of gestation), low birth weight (<2500 grams), small for gestational age for weight (<percentile 10), large for gestational age for weight (>percentile 90), foetal growth (>percentile 90 for weight), and caesarean. Information about the duration of breastfeeding was also obtained (no breastfeeding or less than 16 weeks, ≥16 weeks). Child growth between 0 and 6 months of age was calculated using the age and sex-specific *z*-score weight using the WHO Growth Reference.^23^ Rapid growth was defined as a *z*-score weight gain >0.67 SD, while children with ≤0.67 SD were defined as slow/average growers. More information can be consulted in Valvi.^31^

##### Sociodemographic, lifestyles and dietetic variables at 15 years of age

During the 15-year follow-up visit, adolescents and their mothers filled out a questionnaire administered by trained interviewers. More information about covariates can be consulted in Table S1. The information obtained from the adolescents was: tobacco consumption, subjective physical activity, school canteen attendance at 15 years old, daily meal frequency, frequency of fast-food consumption, and number of siblings. Pubertal development was assessed through the Tanner Stage Scale.^32,33^ This scale evaluates secondary sexual characteristics (Tanner score of breast/genital and pubic hair development) using pictures. More information can be found in Sarzo et al.^34^ and Appendix S1. The global Tanner stage was categorised as stage 5 (postpubescent or adult development) or <5.

The information obtained from the mother in the same follow-up visit was: employment status, education level, tobacco consumption, tobacco consumption of any cohabitant with the adolescent, presence of high BP, high blood glucose, or high cholesterol (yes or no for each condition). The risk of poverty at age 15 was evaluated with the At Risk Of Poverty or Social Exclusion (AROPE) indicator.^35,36^ This indicator is composed of three components: risk of poverty (household income), low work intensity, and severe material deprivation. For the purpose of this study, the AROPE indicator was used as a dichotomic variable (AROPE: yes, no).^36^ Finally, maternal BMI (kg/m^2^: <25, 25–30 [overweight], ≥30 [obesity]) and maternal central adiposity (WHtR ≥ 0.5: yes, no) were assessed using the same procedure as for the adolescent in the same follow-up visit.

##### Dietary variables

Dietary information was obtained from a validated semi-quantitative food frequency questionnaire (FFQ) at the first and third trimesters of pregnancy^37^ and at age 15 answered by the adolescent.^38^ The items in the FFQ had nine possible responses, ranging from ‘never or less than once per month’ to ‘six or more per day’. A standard serving size was assigned to each food item in the FFQ, and this value was transformed into the average daily intake in grams for each participant. We obtained data (expressed in grams per day) for energy-adjusted food groups using the residual method.^39^ The food groups can be consulted in Table S2. For the pregnancy period, adherence to the Mediterranean diet was assessed using the relative Mediterranean Diet Score (rMED) built with dietary information during pregnancy. The rMED was adapted to the pregnant women population by eliminating the alcohol score.^40^ A higher score in this index means higher adherence to the Mediterranean diet.

### Statistical analysis

A descriptive analysis of prenatal and postnatal sociodemographic, dietary, clinical, and lifestyle variables and outcomes was performed. Fisher’s exact test for categorical variables and Kruskal Wallis test for continuous variables were used to detect any differences between the included and non-included populations, and the adolescent´s sex. Bivariate linear regression models were built to study the association between each prenatal/early postnatal and adolescent factor with anthropometric outcomes (zBMI, WHtR, z-waist circumference, z-fat mass), cardiometabolic risk score, zSBP, and zDBP. All the anthropometric measurements and blood pressure are expressed as *z*-scores (except WHtR).

To assess the variables associated with each cardiometabolic risk factor at age 15, multivariable linear regression models were built according to the following process: firstly, core models were built with adolescent (age 15) sociodemographic, clinical, and lifestyle variables associated with a *p* value < 0.20 in the bivariate analysis. The variables in each final core model were selected through a backward elimination procedure, keeping the variables with a *p* value < 0.10^41,42^. Secondly, all food group variables at age 15 were included together in this core model and selected through the same procedure (Model 1). The same process was used to build a core model with prenatal and early postnatal variables (Model 2). The final model (Model 3) was constructed including all variables from Models 1 and 2 together, selecting the variables with a *p* value < 0.10 after a backward elimination procedure.^41,42^ This strategy has been used to strike a balance between retaining relevant variables and minimising unnecessary variables.

To reduce attrition bias, we conducted a complete case analysis and we implemented the inverse probability weighting technique. Firstly, we calculated the weights (the inverse of the probability of selection) for each participant. The stabilised weights were calculated by fitting a multivariable logistic regression model, with ‘participate in the 15-year follow-up’ as the dependent variable. This dichotomic variable has two categories: ‘*participate from baseline to the 15-year follow-up*’ (*n* = 242) and ‘*non-participate in the 15-year follow-up*’ (*n* = 545). As a baseline population, we used the participants in the birth follow-up (*n* = 787). The predictors included in the model were maternal age, parity, parental social class, child sex, maternal country of origin, area of residence, and maternal history of anxiety or depression. The stabilised weights were included in the multivariate regression models of the main analysis.

Statistical analyses were carried out using the R statistical programme version 4.0.3.^43^

## Results

### Prenatal, early postnatal, and adolescent characteristics of the study population

Differences between included and excluded subjects are shown in Table S3. Among the participants, there was a slightly higher percentage of girls, a lower percentage of preterm births, as well as a higher percentage of mothers who presented overweight and obesity before pregnancy than in non-participants.

In our study population, there was a similar percentage of girls (51%) and boys, with a mean age of 15.5 years. At this age, 52% of the participants had reached postpubescent sexual development. Regarding physical activity, almost 40% of the adolescents considered themselves as vigorous/highly active. Around 23% of the participant´s families were AROPE (Table 1). Consumption of most food groups at age 15 was similar in girls and boys, except for a higher vegetable, fruit, and natural juice consumption in girls, while boys consumed more soft drinks (Table 2).

**Table 1:** Characteristics of mother-adolescent pairs included in the study (*n* = 241).

	Mean (SD) or *n* (%) (*n* = 241)		Mean (SD) or *n* (%) (*n* = 241)
Variables at pregnancy		Variables at 15 years old	
Maternal age at conception (years)	30.0 (4.0)	Adolescent’s age (years)	15.5 (0.4)
Maternal country of birth		Sex	
Spain	228 (94.6)	Girl	123 (51.0)
Other	13 (5.4)	Boy	118 (49.0)
Maternal BMI before pregnancy		Number of siblings	
<25	168 (69.7)	0	55 (22.8)
25–30 [overweight]	51 (21.6)	1	154 (63.9)
≥30 [obesity]	22 (9.1)	>1	32 (13.3)
Paternal BMI before pregnancy		Adolescent tobacco consumption	
<25	105 (43.6)	Never	166 (84.7)
25–30 [overweight]	107 (44.4)	Smoke some cigarettes in life	30 (15.3)
≥30 [obesity]	29 (12.0)	Subjective physical activity	
Parity		Sedentary/lightly active	76 (32.2)
Nulliparous	133 (55.2)	Moderately active	62 (28.8)
Multiparous	108 (44.8)	Vigorous/highly active	92 (39.0)
Maternal education level		School canteen assistance	
Up to Primary	68 (28.2)	No	206 (86.9)
Secondary	94 (39.0)	Yes	31 (13.1)
University	79 (32.8)	Meal frequency per day	
Paternal education level		5 per day	162 (64.1)
Up to Primary	97 (40.2)	<5 per day	85 (35.9)
Secondary	96 (39.8)	Frequency of fat food consumption	
University	48 (19.9)	≥Once per week	54 (22.8)
Parental social class		1–3 per month	167 (70.5)
I+II [high]	75 (31.1)	Never	16 (6.8)
III	69 (28.6)	Puberal development (Tanner stage)	
IV+V [low]	97 (40.2)	5	122 (52.4)
Maternal tobacco consumption during		>5	111 (47.6)
No	153 (63.5)	Maternal employment status	
Yes	88 (36.5)	Working	190 (81.5)
Paternal tobacco consumption during pregnancy		Non-working	43 (18.5)
No	134 (55.6)	At risk of poverty or social exclusion (AROPE)	
Yes	107 (44.4)	No	183 (76.6)
Maternal total physical activity during pregnancy (METS)	36.6 (3.6)	Yes	56 (23.4)
Weight gain during pregnancy		Tobacco consumption of any cohabitant	
Recommended	89 (37.4)	No	137 (59.6)
Low	47 (19.7)	Yes	93 (40.4)
High	102 (42.9)	Maternal presence of high blood pressure	
Any glucose impairment during pregnancy		No	202 (86.7)
No	228 (94.6)	Yes	31 (13.3)
Yes	13 (5.4)	Maternal presence of high blood glucose	
**Perinatal and early postnatal variables**		No	219 (94.4)
Preterm (<37 weeks of gestation)		Yes	13 (5.6)
No	229 (95.0)	Maternal presence of high cholesterol	
Yes	12 (5.0)	No	188 (81.0)
		Yes	44 (19.0)
Low birth weight (<2500 g)			
No	230 (95.4)	Maternal BMI	
Yes	11 (4.6)	<25	98 (42.1)
Small for gestational age for weight (<p10)		25–30 [overweight]	75 (32.2)
No	213 (88.4)	≥30 [obesity]	60 (25.8)
Yes	28 (11.6)	Maternal central adiposity (WHtR ≥ 0.5)	
Large for gestational age for weight (>p90)		No	52 (22.3)
No	218 (90.5)	Yes	181 (77.7)
Yes	23 (9.5)		
Foetal growth <p10 for weight			
No	216 (89.6)		
Yes	25 (10.4)		
Foetal growth > p90 for weight			
No	221 (91.7)		
Yes	20 (8.3)		
Caesarean			
No	180 (75.6)		
Yes	58 (24.4)		
Breastfeeding duration			
<16 weeks	80 (33.2)		
≥16 weeks	161 (66.8)		
Growth during the first 6 months of life			
Slow/average	168 (71.2)		
Rapid	68 (28.8)		

*BMI* body mass index, *p10* percentile 10, *p90* percentile 90, *WHtR* waist-to-height ratio.

**Table 2:** Food-group consumption frequency at 15 years old (servings per week).

Foods groups	All		Girls		Boys		*P* val*
	Mean (SD)	Median	Mean (SD)	Median	Mean (SD)	Median	
Dairy products	21.2 (11.4)	19.5	20.8 (10.1)	18.8	21.7 (12.7)	20.1	0.82
Eggs	2.9 (1.6)	3.0	2.9 (1.5)	3.0	2.8 (1.7)	2.9	0.30
Red and white meat	4.6 (2.6)	4.2	4.6 (2.8)	4.1	4.6 (2.4)	4.3	0.62
Processed meat	11.4 (7.0)	10.5	11.9 (7.8)	10.3	11.0 (6.1)	10.6	0.78
Fish and shellfish	6.1(4.2)	5.1	6.0 (3.4)	5.3	6.2 (4.8)	4.8	0.81
Vegetables	12.7 (10.6)	10.3	14.2 (11.5)	11.3	11.2 (9.5)	9.2	0.03
Fruits	17.3 (12.9)	15.3	19.1 (12.6)	16.6	15.4 (12.9)	12.0	0.01
Nuts	3.0 (4.0)	1.7	2.7 (3.5)	1.5	3.5 (4.4)	1.9	0.09
Legumes	1.9 (1.6)	1.4	1.8 (1.5)	1.3	2.0 (1.8)	1.5	0.72
Cereal, pasta and bread	17.4 (8.1)	15.9	17.7 (8.1)	15.9	17.1 (8.1)	15.9	0.68
Potatoes	3.0 (2.0)	2.5	3.0 (1.9)	2.5	2.9 (2.1)	2.6	0.71
Sweets	17.8 (15.5)	13.9	16.9 (13.9)	13.9	18.7 (17.2)	13.9	0.65
Alcoholic beverages	1.5 (4.9)	0.0	1.9 (5.9)	0.0	1.0 (3.4)	0.0	0.31
Animal and vegetable fats	9.9 (9.0)	7.0	11.0 (10.3)	7.1	8.6 (7.1)	6.5	0.23
Fast-prepared-processed food	10.8 (6.0)	9.4	10.5 (5.8)	9.4	11.1 (6.2)	10.0	0.54
Natural juice	1.0 (1.5)	0.5	0.9 (1.1)	0.6	1.1 (1.96)	0.4	0.01
Soft drinks	5.3 (6.6)	3.1	4.3 (5.6)	2.1	6.3 (7.4)	4.1	0.01

*SD* standard deviation.

*p value, comparing food-group consumption frequency between girls and boys using the Kruskal–Wallis test for continuous variables.

Regarding prenatal factors, 9% of the mothers presented obesity before pregnancy, ~36% smoked during pregnancy, and 40% of the families belonged to a low social class. Almost 29% of all children presented rapid growth during the first 6 months of life (Table 1).

### Proportion of overweight and obesity during childhood and other cardiometabolic risk factors at age 15

**Fig. 1:**
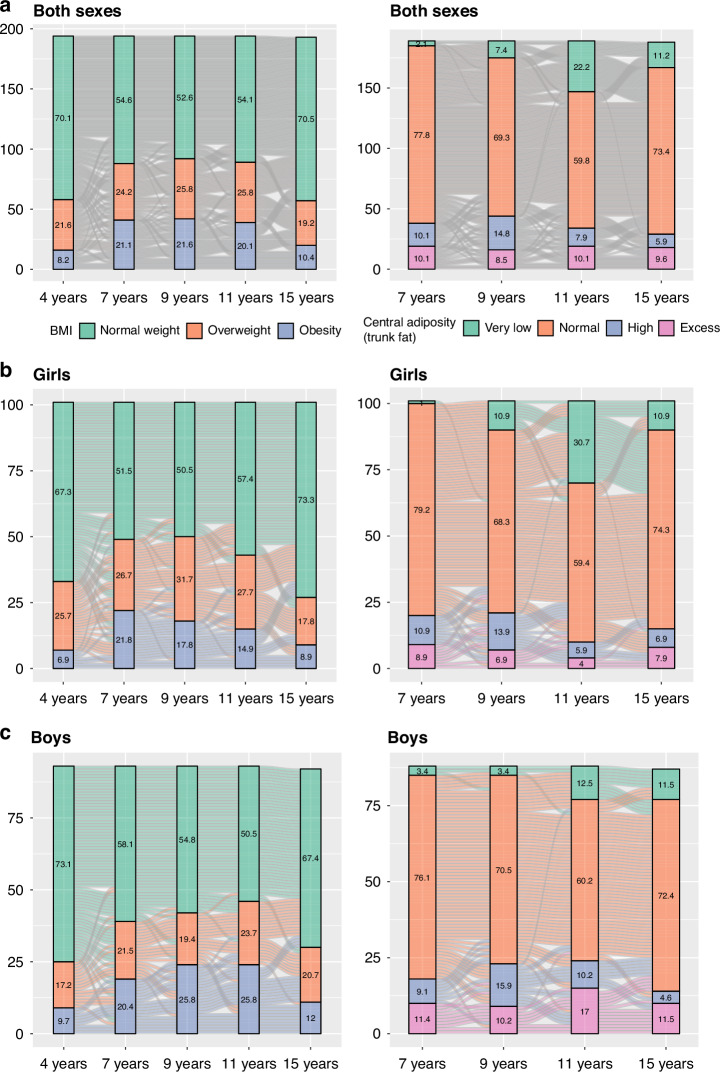
BMI body mass index, WHtR waist-to-height ratio. Proportion of overweight and obesity (OwO) (left) and central adiposity (right) throughout childhood until adolescence in the total sample **A**, girls **B**, and boys **C**. INMA Project (Valencia, Spain).

The proportion of 4-year-old children who presented overweight and obesity (SD > 1 of *z*-score BMI) was 29.8%, which increased up to 47.4% at 9 years of age and then decreased to 29.6% at age 15. At ages 4 and 7, there was an elevated proportion of overweight and obesity in girls, whereas, from age 9 onwards, this trend was higher in boys. Regarding central adiposity, around 20% of children presented high/excess trunk fat (defined as WHtR ≥ 0.50 in boys and ≥0.51 in girls) throughout the follow-up period, observing the highest percentage (23.3%) at age 9. From the age of 9, this percentage is higher in boys than in girls (26.1%, 27.2%, and 16.1% in boys at age 9, 11, and 15, respectively vs. 20.8%, 9.9% and 14.8% in girls at the same ages). The percentage of very low trunk fat is higher in girls than in boys at ages 9 (10.9% vs. 3.4%) and 11 (30.7 vs. 12.5) (Fig. 1). Additionally, around 50% of children and adolescents with overweight presented normal trunk fat at all ages (from 55.4% at age 7 to 47.2% at age 11, Supplementary Fig. S2).

Concerning other cardiometabolic factors in adolescence, 21.3% of the participants presented high blood pressure BP (SBP > 130 mmHg or DBP > 85 mmHg), this percentage was higher in boys. Levels of serum HDL were <40 mg/dL in 9% of the total sample, and 13% of the adolescents presented high levels of serum triglycerides (>150 mg/dl), which was considerably higher in boys (11%) than in girls (2%) (Table 3).

**Table 3:** Cardiometabolic outcomes in adolescents (15 years old).

Anthropometric measures	Total sample	Girls	Boys	*p-*val*
	Mean (SD)	Mean (SD)	Mean (SD)	
BMI (*z*-score)	0.43 (1.16)	0.45 (0.96)	0.41 (1.34)	0.90
Percentage of OwO (BMI *z*-scores +1) % (*n*)	29.46 (71)	26.02 (32)	33.05 (39)	0.26
Waist circumference (cm)	76.00 (10.38)	73.90 (8.26)	78.21 (11.86)	<0.01
Waist-to-height ratio	0.46 (0.06)	0.46 (0.05)	0.46 (0.07)	0.54
WHtR high fat trunk^a^% (*n*)	6.81 (16)	7.44 (9)	6.14 (7)	0.80
WHtR excess fat trunk^a^% (*n*)	11.06 (26)	9.10 (11)	13.16 (15)	0.41
Fat mass (%)	21.51 (8.91)	24.06 (5.91)	15.57 (7.68)	<0.01
**Other cardiometabolic risk factors**				
Systolic blood pressure (mmHg)	118.96 (12.15)	114.41 (9.59)	123.71 (12.75)	<0.01
Diastolic blood pressure (mmHg)	64.33 (7.55)	64.69 (7.35)	63.96 (7.76)	0.36
% high blood pressure^b^	21.28 (509)	8.33 (10)	34.78 (40)	<0.01
Blood HDL-Cholesterol concentrations (mg/dl)	54.39 (11.32)	58.75 (10.73)	50.19 (10.28)	<0.01
Blood LDL-Cholesterol concentrations (mg/dl)	84.65 (23.31)	85.77 (20.13)	83.58 (26.06)	0.05
Blood triglyceride concentrations (mg/dl)	82.17 (47.26)	72.27 (37.13)	91.69 (53.75)	<0.01
Blood glucose concentrations (mg/dl)	92.02 (10.33)	91.13 (10.62)	92.88 (10.03)	0.23
% (*n*) HDL-Cholesterol ≤ 40 mg/dl.	9.00 (18)	4.08 (4)	13.73(149)	0.02
% (*n*) triglycerides ≥ 150 mg/dl	6.50 (13)	2.04 (2)	10.78 (11)	0.01
CMR score	-0.04 (2.79)	-0.05 (2.43)	-0.02 (3.11)	0.59

*BMI* body mass index, *CMR* cardiometabolic risk, *HDL* High-density lipoprotein, *LDL* Low-density lipoprotein, *OwO* overweight and obesity, *SD* standard deviation, *WHtR* waist-to-height ratio.

*p value, comparing cardiometabolic outcomes between girls and boys using Fisher's exact test for categorical variables and Kruskal–Wallis test for continuous variables.

^a^WHtR high-fat trunk is defined as WHtR between 0.50 and <0.53 in boys and 0.51 and <0.54 in girls, and WHtR excess fat trunk is defined as WHtR ≥ 0.53 in boys and ≥0.54 in girls.

^b^High blood pressure is defined as systolic blood pressure >130 or diastolic blood pressure >85 mmHg.

### Prenatal and early postnatal factors associated with anthropometric and other cardiometabolic risk outcomes

**Fig. 2:**
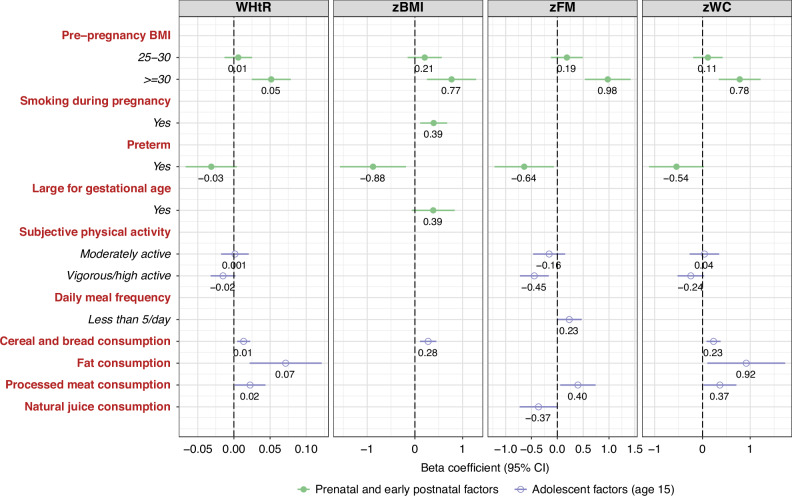
Beta coefficients (95% CI) of the multivariate linear regression between anthropometric measurements of adolescents, and prenatal, early postnatal, and concurrent factors (age 15). INMA Project (Valencia, Spain). zBMI body mass index *z*-score. WHtR waist-to-height ratio. Dietary factors are expressed as 100 g per day.

**Fig. 3:**
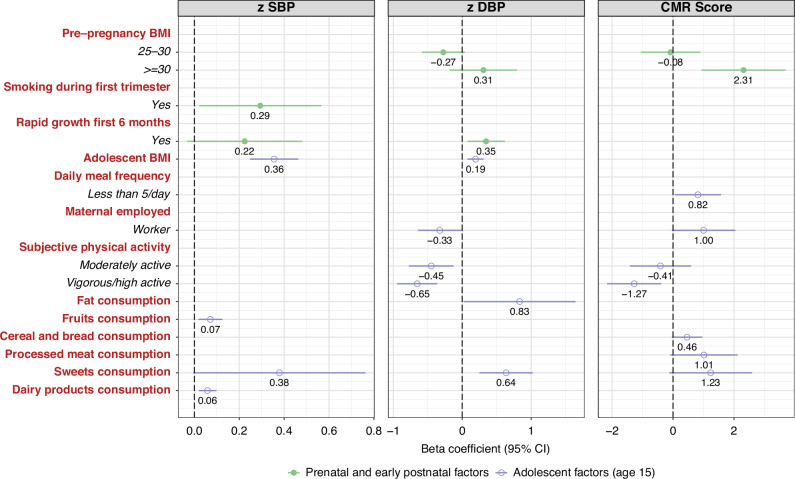
Beta coefficients (95% CI) of the multivariate linear regression blood pressure and cardiometabolic risk score of adolescents, and prenatal, early postnatal, and concurrent factors (age 15). INMA Project (Valencia, Spain). zSBP systolic blood pressure *z*-score, zDBP diastolic blood pressure *z*-score, CMR cardiometabolic risk. Dietary factors are expressed as 100 g per day.

The multivariable linear regression models for the association between prenatal and early postnatal (Model 1) and adolescent factors (Model 2) with the different outcomes are shown in Tables 4 and 5, respectively. Final multivariable models with all factors (Model 3) can be observed in Figs. 2 and 3.

**Table 4:** Multivariate linear regression between concurrent adolescent factors and cardiometabolic risk factors at age 15.

	WHtR		zBMI		zFM		zWC		zSBP		zDBP		CMR score	
	Beta	95% CI	Beta	95% CI	Beta	95% CI	Beta	95% CI	Beta	95% CI	Beta	95% CI	Beta	95% CI
Adolescent BMI (*z*-score)									**0.35**	**0.24, 0.46**	**0.19**	**0.08, 0.30**		
Daily meal frequency (ref. 5/d)														
<5/day			**0.31**	**0.02, 0.61**	**0.29**	**0.047, 0.55**							**0.91**	**0.14, 1.68**
Maternal BMI at age 15 (ref. <25)														
25–30	0.01	−0.01, 0.03	0.22	−0.10, 0.55	0.07	−0.22, 0.35	0.12	−0.16, 0.41						
>30	**0.02**	**0.004, 0.04**	**0.40**	**0.03, 0.76**	**0.41**	**0.09, 0.74**	**0.33**	**0.01, 0.64**						
Maternal employment (ref. non-worker)														
Worker											−0.30	−0.61, 0.01	**1.28**	**0.16, 2.40**
At risk of poverty and/or exclusion (ref. no)														
Yes													0.92	−0.01, 1.86
Subj physical actv (ref. sedentary/light active)														
Moderately active	−0.005	−0.02, 0.01			−0.28	−0.61, 0.04	−0.05	−0.37, 0.27			**−0.47**	**−0.79, −0.15**	−0.50	−1.53, 0.52
Vigorous/highly active	**−0.02**	**−0.04,** **−0.001**			**−0.56**	**−0.85,** **−0.26**	**−0.30**	**−0.59,** **−0.01**			**−0.69**	**−0.97,** **−0.40**	**−1.46**	**−2.34, −0.58**
Cereal, pasta, and bread consumption^a^	**0.01**	**0.01, 0.02**	**0.27**	**0.09, 0.45**			**0.25**	**0.10, 0.40**	0.15	−0.005, 0.31	0.14	−0.01, 0.30	**0.52**	**0.02, 1.03**
Processed meat consumption^a^	**0.02**	**0.013, 0.04**			**0.37**	**0.01, 0.73**	0.34	−0.02, 0.71					**1.19**	**0.08, 2.29**
Fat consumption^a^	**0.07**	**0.02, 0.13**					**0.96**	**0.12, 1.81**	**0.85**	**0.05, 1,65**	**0.97**	**0.15, 1.79**		
Fruit consumption^a^									**0.07**	**0.02, 0.12**				
Dairy product consumption^a^									**0.08**	**0.04, 0.12**				
Nut consumption^a^									0.63	−0.01, 1.28				
Sweet consumption^a^									**0.54**	**0.14, 0.93**	**0.83**	**0.44, 1.22**	**1.44**	**0.07, 2.81**
Natural juice consumption^a^					**−0.39**	**−0.77,** **−0.01**								

INMA Project (Valencia, Spain).

*BMI* body mass index, *CMR* cardiometabolic risk, *DBP* diastolic blood pressure, *FM* fat mass, *SBP* systolic blood pressure, *Subj physical actv* subjective physical activity, *WC* waist circumference, *WHtR* waist-to-height ratio, *z* sex- and-age standardised.

In bold: *p* value from ANOVA *F*-test < 0.05.

^a^Dietary factors are expressed as 100 g per day.

**Table 5:** Multivariate linear regression between prenatal and early postnatal factors and cardiometabolic risk factors at age 15.

	WHtR		zBMI		zFM		zWC		zSBP		zDBP		CMR score	
	Beta	95% CI	Beta	95% CI	Beta	95% CI	Beta	95% CI	Beta	95% CI	Beta	95% CI	Beta	95% CI
Maternal pre-pregnancy BMI (ref. <25)														
25-30	0.01	−0.01, 0.03	0.25	−0.11, 0.60	**0.39**	**0.08, 0.69**	0.23	−0.08, 0.53	−0.07	−0.39, 0.25	−0.29	−0.61, 0.04	0.17	−0.76, 1.09
>30	**0.06**	**0.03, 0.09**	**0.85**	**0.33, 1.37**	**1.20**	**0.76, 1.64**	**0.89**	**0.45, 1.34**	**0.56**	**0.07, 1.05**	**0.77**	**0028, 1.27**	**3.17**	**1.83, 4.51**
Smoking during 1st Tri. of pregnancy (ref. no)														
Yes									**0.33**	**0.04, 0.63**				
Smoking during pregnancy (ref. no)														
Yes			**0.31**	**0.03, 0.60**										
Preterm (ref. no)														
Yes	**−0.05**	**−0.09,** **−0.01**	**−1.11**	**−1.85,** **−0.37**	**−0.72**	**−1.31,** **−0.14**	**−0.89**	**−1.52,** **−0.26**	**−0.77**	**−1.45,** **−0.09**			**−2.77**	**−4.72,** **−0.83**
Large for gestational age (ref. no)														
Yes			0.42	−0.04, 0.88										
Rapid growth during first 6 months of life (ref. no)														
Yes	**0.02**	**0.001, 0.03**	0.29	−0.03, 0.60			**0.31**	**0.04, 0.57**	0.27	−0.01, 0.54	**0.40**	**0.13, 0.68**	**1.31**	**0.50, 2.12**

INMA Project (Valencia, Spain).

*BMI* body mass index, *CMR* cardiometabolic risk, *DBP* diastolic blood pressure, *FM* fat mass, *SBP* systolic blood pressure, *WC* waist circumference, *WHtR* waist-to-height ratio, *z* sex- and-age standardised.

In bold: *p* value from ANOVA *F*-test < 0.05.

Except for zSBP, maternal pre-pregnancy BMI was associated with all anthropometric and other CMR outcomes, even after adjusting by adolescent factors (Model 3, Figs. 2 and 3). Adolescents whose mothers presented obesity before pregnancy showed higher zBMI (*β* [95% CI]: 0.77 [0.25, 1.29], *p* = 0.01), WHtR (*β* [95% CI]:0.05 [0.02, 0.08], *p* < 0.01), zWC (*β* [95% CI]:0.78 [0.34, 1.22], *p* < 0.01), zFM (*β* [95% CI]: 0.98 [0.53, 1.42], *p* < 0.01), zDBP (*β* [95% CI]: 0.31 [−0.18, 0.80], *p* = 0.07), and cardiometabolic risk score (*β* [95% CI]: 2.31 [0.94, 3.69], *p* < 0.01) than those whose mothers were normal weight or overweight.

Tobacco consumption during pregnancy was directly related to zBMI at 15 years of age (*β* [95% CI]: 0.39 [0.11, 0.68], *p* < 0.01), while tobacco consumption in the first trimester of pregnancy was associated with higher zSBP at age 15 (*β* [95% CI]: 0.29 [0.02, 0.57], *p* = 0.03).

Rapid growth during the first 6 months of life was related to higher WHtR, zBMI, zSBP, zDBP, and cardiometabolic risk scores at 15 years of age (Model 2, Table 5); however, these associations only remained for zSBP (*β* [95% CI]: 0.22 [−0.03, 0.48], *p* = 0.9), and zDBP (*β* [95% CI]: 0.35 [0.08, 0.62], *p* = 0.01) when the model was fully adjusted (Model 3, Fig. 3). Finally, adolescents who were born preterm showed lower WHtR (*β* [95% CI]: −0.03 [−0.07, 0.004], *p* = 0.08), zBMI (*β* [95% CI]: −0.88 [−1.57, −0.19], *p* = 0.01), and zFM (*β* [95% CI]: 0.64 [−1.22, −0.07], *p* = 0.03) at age 15.

### Adolescent factors at age 15 associated with anthropometric and other cardiometabolic risk outcomes

As can be seen from Tables 4 and 5, maternal BMI, both during the pre-pregnancy period and at the 15-year-old assessment, was associated with the adolescent’s anthropometric outcomes (Model 1). For example, adolescents whose mothers presented obesity in this follow-up visit presented higher WHtR (*β* [95% CI]: 0.02 [0.004, 0.04], *p* = 0.05), and zFM (*β* [95% CI]: 0.41 [0.09, 0.74], *p* = 0.04). However, when we included both measures simultaneously in the models, only the maternal pre-pregnancy BMI remained statistically significant (Model 3).

The adolescent’s subjective physical activity was statistically significantly related to some anthropometric and other cardiometabolic outcomes, even when the models were adjusted by both prenatal and early postnatal factors. Thus, adolescents who considered themselves physically vigorous/highly active presented lower WHtR (*β* [95% CI]: −0.02 [−0.03, 0.002], *p* = 0.10), lower zFM (*β* [95% CI]: −0.45 [−0.73, −0.17], *p* = 0.01), lower zDBP (*β* [95% CI]: −0.65 [−0.95, −0.36], *p* < 0.01), and lower CMR scores (*β* [95% CI]: −1.27 [−2.16, −0.39], *p* = 0.01) (Figs. 2 and 3).

An adolescent meal frequency <5 per day was associated with higher zBMI, zFM, and cardiometabolic risk score in Model 1, and this factor remained in the final model for zFM (*β* [95% CI]: 0.23 [−0.01, 0.47], *p* = 0.06) and cardiometabolic risk score (*β* [95% CI]: 0.82 [0.06, 1.58], *p* = 0.04).

Regarding the adolescent’s diet, a higher intake of cereals, bread, and pasta was associated with higher WHtR, zBMI, zWC, zSBP, zDBP, and cardiometabolic risk scores (Model 1, Table 4); however, this variable was only kept in the final model for WHtR (*β* [95% CI]: 0.01 [0.004, 0.02], *p* < 0.01), zWC (*β* [95% CI]: 0.23 [0.09, 0.38], *p* < 0.01), and cardiometabolic risk score (*β* [95% CI]: 0.46 [−0.04, 0.96], *p* = 0.07). In the same way, at age 15, processed meat consumption was positively associated with WHtR (*β* [95% CI]: 0.02 [0.001, 0.04], *p* = 0.04), zFM (*β* [95% CI]: 0.40 [0.06, 0.74], *p* = 0.02), and cardiometabolic risk score (*β* [95% CI]: 1.01 [−0.09, 2.12], *p* = 0.07). Finally, higher fat consumption at 15 years of age was directly related to WHtR (*β* [95% CI]: 0.07 [0.02, 0.12], *p* = 0.01), zWC (*β* [95% CI]: 0.92, [0.10, 1.73], *p* = 0.03), and zDBP (*β* [95% CI]: 0.83 [0.02, 1.65], *p* = 0.05), while adolescents with higher consumption of sweets had higher blood pressure (*β* [95% CI]: 0.38 [−0.005, 0.76], *p* = 0.05, for zSBP, and 0.64 [0.25, 1.02], *p* < 0.01, for zDBP) and higher cardiometabolic risk scores (*β* [95% CI]: 1.23 [−0.12, 2.59], *p* = 0.07).

## Discussion

In this longitudinal study, we identified several prenatal and postnatal factors associated with cardiometabolic risk factors evaluated in adolescence. Physical activity, daily meal frequency, and the consumption of some food groups, (i.e. cereals, bread, and processed meat), seem to have an impact on cardiometabolic health at age 15. Additionally, other early-life factors, such as high maternal pre-pregnancy BMI, tobacco consumption during pregnancy, and rapid growth during the first 6 months of life, were independently associated with some of these outcomes, even after adjusting for concurrent adolescent factors. Examining these factors throughout an adolescent’s life enables the identification of at-risk groups to concentrate on health prevention and promotion strategies, thereby mitigating the early onset of excess weight and other cardiometabolic risk factors.

### Proportion and trends of overweight and obesity and central adiposity

In our population, around 29% of 15-year-old adolescents presented as overweight and obese, this percentage being higher in boys than in girls. The results of our study are very similar to those observed in another Spanish study, which reported a prevalence of 29.3% in 12 to 16-year-old adolescents.^44^ Spain is the fifth country in the European Union (EU) with the highest prevalence of overweight and obesity in children and adolescents according to the Global Obesity Observatory.^45^ Nevertheless, in the Health Behaviour in School-aged Children study 2022, the prevalence of overweight and obesity in 15-year-old Spanish children was lower than that observed in our study (23% for boys and 14% for girls).^46^ In the same study, it seems that the prevalence of overweight and obesity in middle and late adolescence could be lower than in early adolescence.

Several studies have analysed the tendency of overweight and obesity throughout childhood. A large Spanish study evaluated the prevalence of overweight and obesity in children from 2 to 17 years of age, observing an increase at 7 years in girls and 9 years in boys, although the trend decreased progressively until 14 years of age.^47^ These observations were similar to those in the present study, except that in our population, the highest peak of overweight and obesity prevalence occurred at age nine in girls and 11 in boys. In the large Health Behaviour in School-aged Children study 2022, which involved several countries in Europe, Canada, and Central Asia, a decrease in the prevalence of overweight and obesity was observed in 11, 13, and 15-year-old children and adolescents, with prevalences of 25%, 22%, and 20%, respectively.^46^ Adolescence is a critical period when different metabolic, hormonal, emotional and behavioural changes occur. These changes in early and mild adolescence, including rapid physical growth,^48^ hormonal changes,^49^ body composition modification,^50^ body image,^51^ and changes in lifestyle behaviours^52^ may have an impact on the BMI trajectories, which could partly explain the decrease in the proportion of overweight and obesity during this period. Nevertheless, this decreasing trend in overweight and obesity at the beginning of adolescence has not been observed in other studies.^53,54^

It is very interesting how the proportion of high/excess trunk fat (central adiposity) is significantly lower than overweight and obesity at all the ages evaluated. The percentage of this excess adiposity is almost a third of that of overweight/obesity assessed by BMI at 11 years of age. Furthermore, a decrease in the proportion of high/excess trunk fat through childhood and adolescence was observed, but it was less pronounced than that observed with BMI. The European Association for the Study of Obesity^55^ and a Commission of Experts have recently redefined the diagnostic of obesity, urging the use of other anthropometric measures that recognise excessive fat accumulation, such as WHtR. While these recommendations target adults, efforts are underway to define cut-off points for detecting excess central adiposity in children and adolescents.^25,56,57^ In accordance with this statement, in our study, about half of the participant categorised as overweight/obese according to BMI had a WHtR corresponding to normal trunk fat. Similar results were observed in the Avon Longitudinal Study of Parents and Children (ALSPAC) study, where more than 50% of the 9 and 15-year-old participants classified into the BMI-overweight category presented very low/normal trunk fat.^24^ These findings highlight the necessity of redefining obesity in the early stages of life based on excess adiposity to establish early and effective prevention strategies.

### Prenatal and early postnatal factors associated with cardiometabolic outcomes

In our study, maternal pre-pregnancy BMI was associated with all outcomes studied (except for SBP), showing a strong relationship with adolescent cardiometabolic risk score. There is consistent evidence about the impact of maternal weight status during children’s early stages of life (conception, pregnancy, and postpartum) on the development of overweight and obesity during childhood. One meta-analysis, which included 79 studies, found that for each increase of 5 kg/m^2^ in maternal pre-pregnancy BMI, the probability of becoming overweight and obese in childhood increased by 55% (OR: 1.55 [95% CI: 1.43–1.69]).^58^ A recent Chinese longitudinal study showed a higher BMI, WC, and percentage of total FM in the 20-year-old offspring of mothers who presented overweight and obesity during pregnancy.^59^

In the present study, we studied the impact of maternal weight status measured at three time points (pre-pregnancy BMI, weight gain during pregnancy, and at the adolescent’s follow-up visit) on adolescent cardiometabolic health. Both pre-pregnancy BMI and concurrent maternal BMI were independently associated with most of the cardiometabolic risks evaluated. However, only maternal pre-pregnancy BMI remained statistically significant when both measures were included in the models. These findings could suggest a stronger effect of pre-pregnancy maternal weight status than concurrent BMI. There are very few longitudinal studies that investigate the effect of maternal weight status at different time points. Similarly to us, Maninno^60^ examined this factor at three time points (pre-pregnancy BMI, gestational weight gain, and maternal BMI at the time of the child’s examination [9–13 years old]). Their results showed that maternal weight status at the three times points was associated with an increased likelihood of childhood obesity during preadolescence, with obesity before pregnancy being the strongest predictor.

The impact of maternal obesity on the cardiometabolic health of their children is complex to understand, however, several hypotheses have been developed; one is related to intrauterine metabolic programming. Evidence from animal studies has shown that maternal obesity during pregnancy was related to changes in the offspring regarding adipose tissue function and adipocyte metabolism,^61^ hypothalamic control of energy balance leading to the development of hyperphagia and obesity,^61^ placental metabolism and function, and epigenetic modifications.^62^ Nonetheless, it is important to recognise that a child’s weight status is also closely and strongly related to their mother’s weight status due to shared behavioural, sociodemographic, and environmental factors, among others. For example, mothers and their children share dietary patterns, observing that children of mothers with obesity have poor-quality diets (high fat, carbohydrates, and sweets),^63^ and differences in feeding and behavioural practices compared with healthy-weight mothers.^64^ As can be seen, the relationship between the pre-pregnancy BMI of the mother and her child is complex and biological, social, environmental, and behavioural aspects must be considered.

Another prenatal factor related to the increase in adolescent BMI was smoking during pregnancy. There is evidence linking tobacco consumption during pregnancy with childhood overweight and obesity. A meta-analysis of 31 observational studies found that children whose mothers smoked during pregnancy had a 37% and 55% increased risk of becoming overweight and obese, respectively.^19^ A posterior large study, with data from 28 European and North American pregnancy and birth cohorts, found a 20% increased risk of childhood overweight (OR: 1.20 [95% CI: 1.03, 1.38]) in children whose mothers were smokers in the first trimester of pregnancy, and a 43% increase (OR: 1.43 [95% CI: 1.31, 1.56]) when mothers continued smoking throughout the entire pregnancy.^65^ In our population, tobacco consumption during the first trimester of pregnancy—but not during the entire pregnancy—was also positively associated with adolescent zSBP, although the effect size was small (*β* [95% CI]: 0.29 [0.02, 0.57]). Agreeing with these results, a recent meta-analysis found that smoking during pregnancy was positively associated with SBP but not DBP.^66^

Finally, in the present study, we have observed higher zDBP at age 15 in those who presented rapid weight gain in the first 6 months of life. Consistent with our results, a longitudinal study conducted in Canada found that accelerated BMI gain between birth and 3 months, and between 3 and 18 months, was associated with higher SBP and DBP in three to 6-year-old children.^67^ Similarly, in the Ugandan EMaBS birth cohort, rapid weight gain from birth to 6 months was significantly related to SBP and DBP in 10- and 11-year-olds.^68^ The evidence suggests that rapid weight gain during this early period of life can impact cardiometabolic health during childhood and adolescence, indicating a window of opportunity for implementing prevention and health promotion strategies.

### Adolescent factors at age 15 associated with cardiometabolic risk outcomes

In our population, several concurrent factors in adolescence were related to the cardiometabolic risk factors evaluated, even after adjusting for prenatal and early postnatal factors. We found that adolescents who ate less than five meals per day presented a higher zFM and cardiometabolic risk score than those who ate five meals per day. Several studies have evaluated the association between daily meal frequency or meal skipping (breakfast or other main meals) and cardiometabolic health outcomes, obtaining similar results.^69,70^ Daily meal frequency and meal skipping have been related to a low-quality diet, such as lower Mediterranean diet adherence,^71^ lower physical activity,^70^ and higher screen time.^70^ These correlations could explain the effect of meal frequency on the adolescent’s cardiometabolic health; however, it is necessary to continue investigating this relationship in-depth to be able to establish precise recommendations for the young population.

Furthermore, as expected, the adolescent’s diet had an impact on cardiometabolic health, however, differences in food group-related outcomes were found. In the present study, cereal, bread, and pasta consumption was directly associated with adolescent WHtR, zBMI, zWC, and cardiometabolic risk scores. A recent systematic review found that the relation between cereal consumption and overweight and obesity in children and adolescents was dependent on the type of cereal consumed, observing a positive association with refined grains and a negative one with whole grain intake.^72^ Another recent study found that the consumption of refined-grain cereal was associated with higher plasma LDL, cholesterol, and triglycerides.^73^ Unfortunately, in our study, this food group included both types of cereals, and we could not analyse the effect separately. Nonetheless, Spain is one of the EU countries with the lowest consumption of whole grain cereals (12 grams per day),^74^ and it is possible that the pattern of cereal consumption was similar in our population study.

Processed meat consumption at age 15 was directly related to WHtR, zFM, and zWC. The results align with several systematic reviews of observational studies, in which a direct relationship was observed between high processed meat consumption and higher BMI, WC,^75^ and risk of metabolic syndrome^76^ in the adult population. In our sample, the median weekly consumption of processed meats among adolescents was around 10 servings, which is much higher than the national recommendation of less than three servings of meat per week, avoiding processed meats.^77^

As expected, the physical activity of the adolescent was related to most of the cardiometabolic health outcomes assessed. Fifteen-year-old boys and girls who considered themselves vigorous/highly active had lower WHtR, zFM, zWC, zDBP, and cardiometabolic risk scores than those who perceived themselves as sedentary/lightly active. Sedentarism and insufficient physical activity are known modifiable factors strongly related to the risk of developing cardiovascular and metabolic risk factors and diseases throughout childhood, adolescence, and adulthood.^12,78,79^ For example, in a recent analysis conducted with participants from the ALSPAC, each 1-min of sedentary time was positively associated with systolic and diastolic blood pressure^80^ and FM,^12^ meanwhile light physical activity was related with a decrease of these cardiometabolic risk factors, and also LDL cholesterol, triglyceride and total cholesterol^81^ in childhood, adolescence, and young adulthood. Also, the latest systematic review suggested that physical activity during childhood and adolescence could have a protective effect against developing multimorbidity in adulthood.^82^ In our study, we evaluated physical activity based on adolescents’ self-reports. Despite being a subjective measure, this variable was linked to the assessed health outcomes. This information could be used as a simple method to detect at-risk adolescent populations, where physical-activity-based interventions and programmes can be effectively focused.

This study has several limitations. The most important is the loss of participants from the prenatal period to 15 years of age. These losses of follow-up, common in long-term cohort studies, may result in attrition bias and a decrease in sample size, affecting the study’s representativeness. To minimise this bias, we performed the main analyses using the inverse probability weighting technique. Nevertheless, it is possible that selection bias may not be fully mitigated, and the results should be interpreted with caution and confirmed by further studies. Another limitation is the use of subjective information as a proxy to evaluate certain characteristics of the population, such as the physical activity of adolescents. Nonetheless, there is evidence of the correlation between self-reported and objective measurements of physical activity.^83^ Furthermore, the components of the cardiometabolic risk score have been standardised using the study population itself as a reference population. This is because there are currently no standardised *z*-scores in a standard reference population for the age of the study participants. This makes it difficult to compare the results with other populations. Finally, this study has analysed multiple sociodemographic, clinical, dietary, and lifestyle variables, both prenatal and postnatal, nonetheless, there is a lack of information on other factors that could impact the development of cardiometabolic risk factors in adolescents, such as sleep quality or screen time.

The major strength of our study is its prospective nature, which has allowed the provision of extensive information, from the prenatal period to adolescence, regarding sociodemographic, dietary, and lifestyle characteristics that could affect cardiometabolic health. Another strength is the quality of the data used. The information was gathered by trained fieldworkers using validated questionnaires and standardised protocols, which helps to reduce information bias in the data collected.

## Conclusion

This longitudinal study has identified various modifiable factors, such as physical activity, daily meal frequency, and the consumption of cereals and processed meats, which are associated with cardiometabolic health in adolescents. Furthermore, maternal factors during pregnancy, such as maternal pregestational BMI or tobacco consumption during pregnancy, seem to significantly impact the development of cardiometabolic risk factors even during adolescence. Deepening our knowledge of factors that contribute to the development of cardiometabolic risk factors at young ages could improve the identification of susceptible populations. Thus, effective prevention, health promotion, and management interventions could be implemented, not only during adolescence but also in the very early stages of life, such as during prenatal periods. More longitudinal and multicentric studies are needed to understand the complex relationship of factors that have an impact on cardiometabolic health.

## Supplementary information

Supplementary Material

## Data Availability

The datasets generated during and/or analysed during the current study are available from the corresponding author on reasonable request.
