# Supplementary material for: Determinants of overweight and obesity and other cardiometabolic risks in adolescents: a Spanish longitudinal birth study
Source: Pediatr Res. 2025 Jul 18;98(6):2087–100. doi: 10.1038/s41390-025-04273-w (PMC12811136; doi:10.1038/s41390-025-04273-w)

Supplemental Material

Determinants of obesity and other cardiometabolic risks in adolescents: A Spanish Longitudinal Birth Study

Manuel Lozano, Jorge Vallejo-Ortega, Natalia Marín, Llúcia González-Safont, Ana Esplugues, Maria-Jose Lopez-Espinos, Andrea Beneito, Sandra González-Palacio, Sabrina Llop, Raquel Soler-Blasco

Figure S1. Flow chart describing the process of selecting participants in the INMA Project to be included in the present analysis.

Appendix S1. Methodology

Table S1. Information about prenatal and postnatal sociodemographic, clinical lifestyle and dietetic covariables used in the study.

Table S2. Specific food items included in each food group from FFQ administered in pregnancy and at age 15.

Table S3. Sociodemographic and environmental characteristics of study participants, and differences between populations included and excluded in the study. INMA Project

Figure S2. Proportion central adiposity throughout childhood until adolescence in participants who presented normal and excess weight. INMA Project (Valencia, Spain).

Figure S1. Flow chart describing the process of selecting participants in the INMA Project to be included in the present analysis.

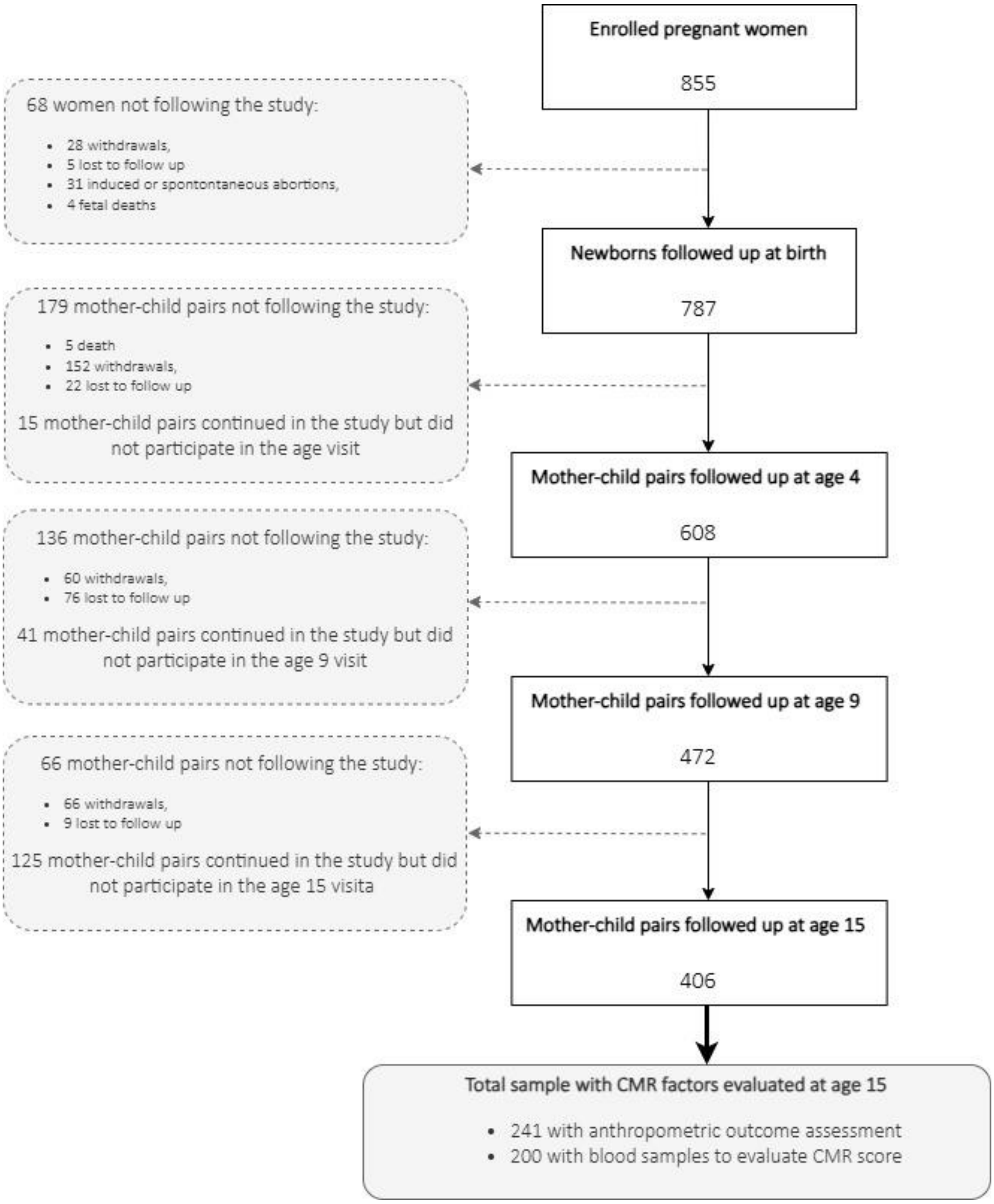

Appendix S1. Methodology

Clinical assessment

a) Anthropometry assessment

Height was measured without shoes with a mobile stadiometer (Seca model 213) with a precision of 1 mm.

Weight and fat mass was measured barefoot and in light clothing using an electronic scale (Tanita model BC-351) with a precision of 0.05 kg. and 0.1%, respectively.

Waist circumference was measured following the WHO protocol [1,2] at ages 7, 9, 11 and 15 standing, relaxed, with the arms at the sides, and feet about 25 cm apart, in the middle point between the lower margin of the last palpable rib and the top of the iliac crest after a normal expiration, using an inelastic tape (Seca 201) with a precision of 1 mm.

Blood pressure (BP) was measured during the clinical examination in the same follow-up visit using a standardized protocol: after at least 5 minutes of rest in sitting position with both feet flat on the floor, the appropriately sized sphygmomanometer cuff (14-24 cm of diameter) was fitted preferably on the adolescent's right arm, at the height of the heart. The arm was relaxed, without clothing that compressed it, supported on the table. Three consecutive measurements were taken by oscillometric device (OMROM M4-I) with at least one-minute time intervals between measurements. During the procedure, the adolescent was relaxed and silent. The device has an accuracy of ±3 mmHg and it is clinically validated according to the International Protocol of the European Society of Hypertension and the criteria suggested by the British Hypertension Society for its use in children and adolescents [3]. Systolic and diastolic blood pressures (SBP and DBP) from the three measurements were recorded and the mean of the second and the third measurements was calculated and used in further analysis. Median blood pressure (MBP) was calculated using the formula (SBP +DBP)/3 + SBP.

b) Pubertal development assessment

Pubertal development was assessed by mean of the Tanner Stage Scale [4,5]. This scale evaluates secondary sexual characteristics (Tanner score of breast/genital and pubic hair development) using pictures. After receiving an explanation from a trained nurse, the adolescent were shown five images representing the five Tanner stages, ranging from 1 (prepuberty or puberty not initiated) to 5 (post-puberty or adult development), and they chose one. The global Tanner stage was calculated as the mean of two scores (rounded up) and then, categorised in stage 5 or <5.

REFERENCES

1. World Health Organization. *Waist Circumference and Waist-Hip Ratio: Report of a WHO Expert Consultation*.; 2011.
2. World Health Organization. *WHO STEPwise Approach to Surveillance*.; 2008.
3. Stergiou GS, Parati G, Asmar R, O'Brien E. Requirements for professional office blood pressure monitors. *J Hypertens*. 2012;30(3):537-542. doi:10.1097/HJH.0b013e32834fcfa5.
4. Marshall WA, Tanner JM. Variations in the pattern of pubertal changes in Girls. *Arch Dis Child*. 1970;45(239):13-23. doi:10.1136/adc.45.239.13
5. Marshall WA, Tanner JM. Variations in the Pattern of Pubertal Changes in Boys. *Arch Dis Child*. 1970;45(13):13-23.

Table S1. Information about prenatal and postnatal sociodemographic, clinical lifestyle and dietetic covariables used in the study.

Variable	Categories
**Sociodemographic, lifestyle and dietetic variables during pregnancy and birth**	
Maternal BMI before pregnancy	Low and healthy weight [<25 kg/m2], overweight [25 ≤ 30], and obesity [≥30]
Paternal BMI before pregnancy	Low and healthy weight [<25 kg/m2], overweight [25 ≤ 30], and obesity [≥30]
Maternal education level	Up to primary, secondary, university
Paternal education level	Up to primary, secondary, university
Maternal working status at pregnancy	Non-worker, worker
Paternal working status at pregnancy	Non-worker, worker
Parental social class during pregnancy	I+II [high], III, IV+V [low]
Maternal tobacco consumption during 1st trimester of pregnancy	Yes, no
Maternal tobacco consumption during whole pregnancy	Yes, no
Parity	0, ≥1
Maternal total physical activity during pregnancy	Continuous: expressed as overall metabolic equivalent of task levels
Weight gain during pregnancy	Recommended, low, high
Any glucose impaired during pregnancy	Including impaired glucose tolerance, gestational diabetes or diabetes diagnosis prior to pregnancy.<br>Yes, no
Child's sex	Girl, boy
Preterm (<37 week of gestation)	Yes, no
Low birthweight (<2500 grams	Yes, no
Small for gestational age for weight<br>(< percentile 10)	Yes, no
Large for gestational age for weight<br>(> percentile 90)	Yes, no
Caesarean	Yes, no
Breastfeeding	No breastfeeding or less than 16 weeks, ≥16 weeks
Rapid growth between 0 to 6 months of age	Calculated using the age and sex specific z-score weight using the World Health Organization Growth Reference.<br>Yes, no
**Sociodemographic, lifestyles and dietetic variables at 15 years of age**	
Adolescent tobacco consumption	"*Have you smoked a cigarette in your life?*"<br>Yes, no

Variable	Categories
Subjective physical activity	"*Globally, considering all your physical activity, how do you consider yourself?*"<br>Sedentary/light active, moderately active, vigorous/high active
School canteen attendance	Yes, no
Daily meal frequency	5 per day, <5 per day
Frequency of fast-food consumption	never, 1-3 per month, ≥ once per week
Number of siblings	none, 1, >1
Pubertal development	From Tanner Stage Scale (see Appendix S1)<br>Stage 5 (post-puberty or adult development) or <5
Maternal working status	Non-worker, worker
Maternal education level	Up to primary, secondary, university
Maternal tobacco consumption	Yes, no
Tobacco consumption of any cohabitant with the adolescent	Yes, no
Maternal presence of high blood pressure	Yes, no
Maternal presence of high blood glucose	Yes, no
Maternal presence of high cholesterol	Yes, no
Risk of poverty	Evaluated with the At Risk Of Poverty or social Exclusion (AROPE) indicator.<br>Yes, no
Maternal body mass index	<25, 25-30 [overweight], ≥30 [obesity]
Maternal central adiposity (WHtR≥0.5)	Yes, no

Table S2. Specific food items included in each food group from FFQ administered in pregnancy and at age 15.

	Pregnancy	15 years old
**Dairy products**	Whole, semi-skimmed, and skimmed milk, condensed milk, cream or milk cream, whole yogurt, skimmed yogurt, cottage cheese, white or fresh cheese, cured, semi-cured or creamy cheese, custard, flan, pudding, ice cream	Idem
**Eggs**	Eggs	Idem
**Red and white meat**	Chicken, game meat: (rabbits, quail, duck), beef, pork, lamb, liver, organ meats, hamburger	Idem
**Sausage**	Sausage, bacon, pâtés	---
**Processed meat**	---	Serrano or York ham, salchichón, chorizo, salami, mortadella, fuet, sobrasada, sausages, pâtés and hamburgers
**Fish and shellfish**	Lean fish, blue fish, canned fish, fish croquettes, clams, mussels, oysters, squid, octopus, seafood.	Idem
**Vegetables**	Spinach, cabbage, cauliflower, broccoli, lettuce, endive, tomato, onion, carrot, pumpkin, green beans, eggplant, zucchini, cucumbers, peppers, artichokes, asparagus, garlic	Idem
**Fruits**	Oranges, tangerines, banana, apple, pear, peach, nectarine, apricot, watermelon, melon, grapes, plums, kiwi, olives, orange juice	Idem
**Nuts**	Almonds, peanuts, pine nuts, hazelnuts	Idem
**Legumes**	Lentils, chickpeas, pinto or white beans.	Idem
**Cereal, pasta and bread**	Breakfast cereals, boiled corn, cooked rice, spaghetti, noodles, macaroni and similar, white and wholemeal bread	Idem
**Potatoes**	French Fries, boiled, roasted potatoes, bag of chips	Idem
**Sweets**	Chocolate, added sugar, jams, honey, biscuits, buns, cookies, pastries	Idem
**Alcoholic beverages**	Wine, beer, dry wines, liqueurs, gin, vodka etc	----
**Animal fat**	Butter	Butter, olive oil, other vegetable oils, mayonnaise
**Vegetable fat**	Olive oil, other vegetable oils, mayonnaise	
**Prepared food**	Pizza, vegetable soup or puree, chicken or ham croquettes, ketchup	----
**Fast-prepared-processed food**	----	Meat derivatives (nuggets, croquettes, fingers, San Jacobo), fish derivatives (surimi, hake sticks), bag of chips, bags of popcorn, corn crusts, cones, pizza, mayonnaise, ketchup and tomato sauce
**Natural juice**	---	Freshly squeezed natural juice
**Soft drinks**	---	Sugary soft drinks, bottled juices, Nectars and other juice drinks, sugary energy drinks, sweetened soft drinks, sweetened energy drinks

Table S3. Sociodemographic and environmental characteristics of study participants, and differences between populations included and excluded in the study. INMA Project

	Included population (n= 241)	Non- included population[a] (n= 546)	
	N (%)	(%)	p-value[b]
Maternal age at conception (years) [c]	30.0 (4.0)	29.7 (4.5)	0.63
Maternal country of birth			
Spain	228 (94.6)	466 (85.3)	
Other	13 (5.4)	80 (14.7)	
Maternal BMI before pregnancy			
<25	168 (69.7)	397 (72.8)	0.06
25-30 [overweight]	51 (21.2)	95 (17.4)	
≥30 [obesity]	22 (9.1)	53 (9.7)	
Paternal BMI before pregnancy			
<25	105 (43.6)	244 (44.9)	0.53
25-30 [overweight]	107 (44.4)	240 (44.1)	
≥30 [obesity]	29 (12.0)	60 (11.0)	
Parity			
Nulliparous	133 (55.2)	300 (54.9)	0.80
Multiparous	108 (44.8)	246 (45.1)	
Maternal education level			
Up to primary	68 (28.2)	199 (36.4)	0.07
Secondary	94 (39.0)	241 (44.1)	
University	79 (32.8)	106 (19.4)	
Paternal education level			
Up to primary	97 (40.2)	268 (49.4)	0.76
Secondary	96 (39.8)	208 38.4)	
University	48 (19.9)	66 (12.2)	
Parental social class			
I+II [high]	75 (31.1)	96 (17.6)	0.96
III	69 (28.6)	146 26.7)	
IV +V [low]	97 (40.2)	304 55.7)	
Maternal tobacco consumption during			
No	153 (63.5)	307 (57.0)	0.42
Yes	88 (36.5)	232 (26.9)	
Weight gain during pregnancy			
Recommended	89 (37.4)	188 (36.8)	0.55
Low	47 (19.7)	111 (20.6)	
High	102 (42.9)	241 (44.6)	
Sex			
Girl	123 (51.0)	370 (47.0)	<0.01
Boy	118 (49.0)	417 (53.0)	
Preterm (< 37 weeks of gestation)			
No	229 (95.0)	510 (93.6)	0.002
Yes	12 (5.0)	35 (6.4)	
Large for gestational age for weight (>p90)			
No	218 (90.5)	483 (89.4)	0.48
Yes	23 (9.5)	58 (10.6)	
Growth at first 6 months of life			
Slow/average	168 (71.2)	307 (68.2)	0.02
Rapid	68 (28.8)	143 (31.8)	

[a]Mother–child pairs included in the cohort at birth but not participating in the present study for different reasons (deaths, withdrawals, lost to follow-up, unavailability anthropometric measurements at 15-years-old visit).

[b]p-value, comparing women's characteristics between included and non-included population using Fisher's Exact Test for categorical variables and Kruskal Wallis Test for continuous variables.

Figure S2. Proportion central adiposity throughout childhood until adolescence in participants who presented normal and excess weight. INMA Project (Valencia, Spain).

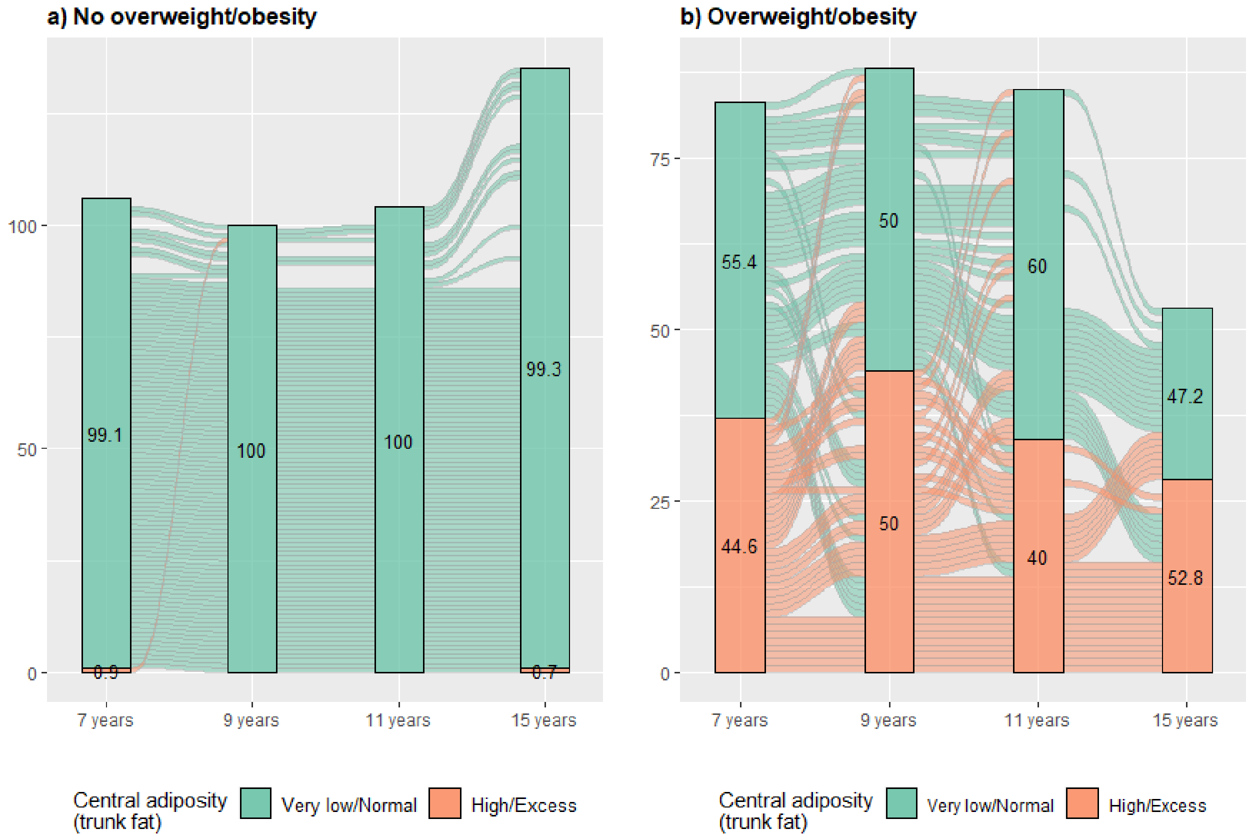

Supplement: Supplementary file 1 — Supplementary Material [file 41390_2025_4273_MOESM1_ESM.pdf]
